# Integrated Proteomics and Metabolomics Profiling Unravels Molecular Mechanisms Underlying Postmortem Meat Quality Between Two Ages and Muscle Types in Sansui Duck

**DOI:** 10.3390/ani15192773

**Published:** 2025-09-23

**Authors:** Jinjin Zhu, Ai Liu, Jiying Wen, Baoguo Zhu, Yifu Rao, Biqiong Yao, Surintorn Boonanuntan, Shenglin Yang

**Affiliations:** 1 Key Laboratory of Animal Genetics, Breeding and Reproduction in the Plateau Mountainous Region, Ministry of Education, Guizhou University, Guiyang 550025, China; jinjinz1230@163.com (J.Z.); liuai4735@163.com (A.L.); wenjiying1221@outlook.com (J.W.); zhubg24@gmail.com (B.Z.); m18334067492@163.com (Y.R.); 2 Agricultural and Rural Bureau of Sansui County, Sansui County 556500, China; yaobiqiong99@163.com; 3 School of Animal Production and Innovation, Suranaree University of Technology, Nakhon Rachasima 30000, Thailand; surinton2012@gmail.com

**Keywords:** proteomics, metabolomics, meat quality changes, mechanism, Sansui duck

## Abstract

As a major source of high-quality protein in China, duck meat such as the renowned Beijing Duck plays a critical role in the poultry industry. Sansui duck, a prized native breed, is valued for its tender meat and rich flavor, yet molecular mechanisms underlying its meat quality remain poorly studied. This study employed metabolomics and proteomics techniques to conduct a comprehensive comparative analysis of the breast and thigh muscles from 90-day-old (90X, 90T) and 468-day-old (468X, 468T) Sansui ducks. The meat quality traits indicated that the shear force and redness (a*) were significantly higher in the 468T and 468X groups compared to the 90X and 90T groups (*p* < 0.05). Similarly, the shear force values of the 90T and 468T groups were significantly higher than those of the 90X and 468X groups (*p* < 0.05). Quantitative proteomics analysis revealed differentially expressed proteins (DEPs) significantly enriched in oxidative phosphorylation and ribosomal biogenesis pathways. Non-targeted metabolomics identified differentially expressed metabolites (DEMs) concentrated in amino acid and lipid metabolism pathways. Correlation analysis indicated that in the comparison between 90X and 468X, 18 DEPs and 10 DEMs were closely associated with fleshiness, whereas in the comparison between 468X and 468T, 23 DEPs and 19 DEMs were closely associated with fleshiness. Integrating proteomics and metabolomics data analysis, proteins such as A0A8B9TTI1, R0JRM6, and A0A8B9SQI5, along with metabolites including L-lysine, L-pyrrolidone, and γ-aminobutyric acid from lysine degradation, butanoate metabolism, and 2-oxocarboxylic acid metabolism pathways, can be proposed as key factors influencing meat quality through pathways including lysine degradation, butanoate metabolism, and 2-oxocarboxylic acid metabolism in older ducks. In contrast, the protein R0JXJ3 and metabolites choline and L-glutamine may determine meat quality differences between anatomical sites through the ABC transporter pathway. These findings provide molecular insights and potential biomarkers for genetic breeding and meat quality improvement in Sansui ducks.

## Simple Summary

Sansui duck is renowned for its high-quality meat; however, further genetic improvement in its meat quality remains a challenge. In this study, we combined quantitative proteomics and untargeted metabolomics to analyze breast and thigh muscles from Sansui ducks at different developmental stages. We identified key differentially expressed proteins and metabolites strongly associated with meat quality traits. These findings offer candidate biomarkers and insights for the future enhancement of meat quality in Sansui ducks.

## 1. Introduction

Duck meat is a vital source of high-quality protein in the diets of many countries, and its quality characteristics directly influence market competitiveness and consumer acceptance of related products [1,2]. Sansui duck, a highly regarded indigenous Chinese poultry breed, is recognized as a National Geographical Indication protected product and serves as a leading local industry. Its meat is highly prized for its tenderness, and it is also renowned for its distinctive flavor and rich cultural significance [3]. Furthermore, Sansui duck meat is characterized by a high proportion of essential amino acids and a low fat content, thereby meeting contemporary consumers’ preferences for healthier meat products. Its strong environmental adaptability and disease resistance further provide advantageous conditions for large-scale farming. To summarize, Sansui duck can be regarded as a strategic resource for ensuring biodiversity and agricultural security. In addition, it can be considered a distinctive pillar of regional economic development and a vehicle for the preservation of intangible cultural heritage. This indicates promising prospects for future development.

The quality of duck meat is influenced by multiple factors, with age and muscle cut being among the most significant determinants. Meat quality is a multidimensional and comprehensive index system that includes key attributes such as tenderness, color, water-holding capacity, and the composition of flavor compounds [4]. Among these, shear force is a primary indicator of meat tenderness and directly influences the eating texture. Dynamic changes in pH are closely associated with water retention and the rate of spoilage. Meat color parameters (L*, a*, b*) significantly influence consumers’ initial purchasing decisions through visual perception, which is governed by the oxidation state of myoglobin and cytochrome metabolism [5]. Research has demonstrated that both animal age and muscle type are critical factors affecting meat quality. As animals age, increased collagen cross-linking within muscle tissues can reduce tenderness. Additionally, variations in intramuscular fat deposition contribute to differences in flavor compound accumulation. Furthermore, the distinct energy metabolism pathways and muscle fiber compositions—fast-twitch fibers in breast muscles versus slow-twitch fibers in thigh muscles—lead to significant differences in meat quality characteristics [6,7]. The 90-day-old ducks are in the late middle growth phase, during which muscle and skeletal development is largely complete. This stage is typical for commercial slaughter, and the meat quality at this age represents the characteristics of mainstream market products. In contrast, the 468-day-old ducks are in the late adult stage, having undergone full physiological maturation. At this stage, their muscle tissues are fully developed, and the accumulation of connective tissues and flavor compounds has reached a stable state [8]. Evidence has been established indicating that age significantly influences the deposition of flavor compounds and nutritional constituents in poultry meat [9]. Specifically, in geese, the contents of total essential amino acids (TEAAs) and polyunsaturated fatty acids (PUFAs) increase with advancing age, thereby enhancing the nutritional value of the meat [10]. Similar age-related compositional changes have been observed in duck meat. For instance, breast muscle from D900 ducks exhibits higher amino acid content compared to D60 ducks, particularly in aspartic acid (Asp), associated with umami taste, and threonine (Thr), which contributes to sweetness [8]. In the context of research on duck meat, the breast and thigh muscles are among the most frequently analyzed muscles.

In recent years, the rapid advancement of omics technologies has offered new insights into the molecular mechanisms underlying meat quality formation. Among these, the integration of proteomics and metabolomics has emerged as a central approach in related research. Proteomics investigates the entire set of proteins in muscle tissue, enabling the systematic identification of dynamic changes in differentially expressed proteins (DEPs). This approach is particularly effective in identifying key functional proteins that influence meat quality, such as enzymes involved in energy metabolism, antioxidant proteins, and structural proteins of muscle fibers. The expression or post-translational modification of these proteins directly affects muscle energy supply, oxidative stability, and muscle fiber toughness, acting as “upstream regulators” of meat quality traits [11,12]. For instance, Zhang et al. (2018) utilized proteomics analysis to identify 21 differentially expressed proteins between meat samples with varying drip loss levels, including structural proteins and metabolic enzymes, whose expression profiles were significantly correlated with muscle water-holding capacity [13]. In a similar vein, Fuente et al. (2019) employed proteomics techniques to detect five significantly differentiated protein bands (*p* < 0.05) that effectively distinguished normal meat from dry, firm, and dark (DFD) meat, highlighting their potential as biomarkers for pre-slaughter stress assessment in cattle [14]. In contrast, metabolomics focuses on variations in the abundance of small-molecule metabolites. Its strength lies in capturing the characteristics of “metabolic flux” during meat quality development—that is, the balance among substrate consumption, the accumulation of intermediate metabolites, and the production of end products. For example, changes in metabolites involved in amino acid, lipid, and nucleotide metabolism are closely associated with meat quality attributes such as umami, juiciness, freshness, and pH value [15,16]. Previous studies have demonstrated that lactate accumulation in livestock and poultry meat is strongly linked to postmortem pH decline, while the concentrations of metabolites such as inosine monophosphate and free amino acids directly determine umami characteristics [9,17]. Subbaraj et al. (2016) employed HILIC-MS technology to elucidate the association mechanism between polar metabolites and myoglobin stability in mutton, confirming a positive correlation between antioxidant content and meat color stability [18]. Moreover, multi-omics integration enables causal analysis of protein–metabolite relationships [19] and the identification of key regulatory networks that would remain obscured if either approach were used in isolation. For instance, Gu et al. (2024) identified age-specific biomarkers related to purine metabolism in duck muscle using integrated multi-omics data, providing key insights into the mechanisms of meat quality changes during aging [20].

However, systematic investigations into the synergistic effects of age and muscle type on meat quality remain limited for the indigenous Sansui duck breed. In particular, the intrinsic regulatory mechanisms underlying meat quality attributes have not yet been elucidated from combined proteomics and metabolomics perspectives. In this study, breast (90X) and thigh (90T) muscles from 90-day-old ducks, as well as breast (468X) and thigh (468T) muscles from 468-day-old ducks, were selected to assess their physical properties. Integrating untargeted metabolomics with data-independent acquisition (DIA)-based quantitative proteomics, this study conducted a systematic comparative analysis of samples in different physiological states, aiming to elucidate the molecular mechanisms underlying differences in meat quality traits and identify relevant candidate biomarkers, thereby providing a theoretical foundation for subsequent research. Further validation through larger-scale population studies and in-depth functional experiments will help confirm the practical application value of these findings, which is of great significance for promoting their translation into precision breeding and meat quality improvement strategies in the Sansui duck industry.

## 2. Materials and Methods

### 2.1. Animal Sample Collection

Twenty pure female Sansui ducks (*Anas platyrhynchos*) were randomly selected from Guizhou Sansui Wangping Agriculture & Animal Husbandry Co., Ltd. (Sansui, China), including ten D90 ducks (1.13 ± 0.071 kg) and ten D468 ducks (1.45 ± 0.098 kg). All ducks at Guizhou Sansui Wangping Agriculture and Animal Husbandry Co., Ltd. (GSWA) were raised under standardized farming conditions, utilizing a floor rearing system. Throughout the rearing period, the ducks were provided with ad libitum access to commercial compound feed and drinking water. The husbandry management strictly adhered to standardized production protocols. The composition and nutritional level of the basic diet is shown in Tables S1 and S2. This experiment was reviewed and approved by the Guizhou University Sub-Committee of Experimental Animal Ethics (Guiyang, China; No. EAE-GZU-2022-E032).

### 2.2. Tissue Collection

Ducks with apparent good health, absence of physical abnormalities, conformity to standard breed weights for their age, and genetic consistency as per the supplier’s records were selected for each experimental group. Following a 12 h fasting period, experimental ducks were humanely euthanized via cervical exsanguination combined with sedative pretreatment (sodium pentobarbital, 35 mg/kg body weight administered intravenously through the metatarsal vein). Then, breast muscle (pectoralis major) and thigh muscle (femorotibialis) were aseptically excised from the left carcass side. The entire process of muscle dissection, sampling, and flash-freezing was completed within 15 min for each individual duck. Muscle samples were divided into three aliquots: One portion was immediately analyzed for meat quality, including shear force and meat color parameters. Another portion was stored at 4 °C (HYC-4103D, Haier, Qingdao, China) for 24 h for pH measurement. The remaining sample was immediately flash-frozen in liquid nitrogen and subsequently transferred to a −80 °C refrigerator (DW-86 L626, Haier, China) for subsequent proteomics and metabolomics analyses. To standardize statistical nomenclature, muscle specimens were systematically coded as follows: D90X: pectoral muscle from 90-day-old ducks; D90T: thigh muscle from 90-day-old ducks; D468X: pectoral muscle from 468-day-old ducks; D468T: thigh muscle from 468-day-old ducks.

### 2.3. Meat Quality Determination

#### 2.3.1. pH Value Determination

The pH values of the pectoralis major and thigh muscles were measured at 24 h postmortem using a pH-star meter (Matthäus GmbH, Eckelsheim, Germany). Prior to measurement, the pH probe was calibrated with standard buffer solutions (pH 4.0 and 7.0, certified by NIST). Prior to measurement, all muscle samples were equilibrated to room temperature (20 °C ± 5 °C). The probe was fully inserted into the muscle tissue, and readings were recorded once stable values were displayed on the digital screen. Triplicate measurements were taken at three randomly selected points on each sample, and the mean value was calculated [21].

#### 2.3.2. Shear Force Measurement

Muscle samples (2 cm × 1 cm × 1 cm) were excised parallel to the myofiber orientation using a standardized template from three random points. Shear force was determined using a TA.XT Plus texture analyzer (Stable Micro Systems, Godalming, UK) equipped with a Warner-Bratzler shear blade (1.0 mm thickness). Prior to analysis, the instrument was force-calibrated using a certified 5 kg standard weight. The blade traversed perpendicular to the myofiber direction at a crosshead speed of 200 mm/min under a 15 kg load cell. The entire measurement process was completed at room temperature of 20 °C ± 5 °C. Triplicate measurements per sample were performed, and the mean peak force (N) was calculated [22].

#### 2.3.3. Color Values Measurement

Meat color parameters (L* [lightness], a* [redness], and b* [yellowness]) of pectoralis major and femoralis muscles were measured using an Opto-Star colorimeter (Matthäus GmbH, Eckelsheim, Germany). The total color difference (∆E) can be calculated from these. Prior to analysis, the instrument was calibrated with a certified calibration module. The surface of each muscle sample was carefully trimmed to remove any visible connective tissue, epimysium, and subcutaneous fat. Three random locations per sample were measured after stabilization of the displayed values, with the results expressed as mean values.

### 2.4. Data-Independent Acquisition Proteomics Analysis

#### 2.4.1. Protein Extraction and Digestion

Three biological replicates were randomly selected per experimental group (*n* = 3). These samples were pulverized in liquid nitrogen and lysed with SDT buffer (containing 1% DTT and 100 mM NaCl) followed by ice-bath ultrasonication. After heat denaturation at 95 °C, the supernatant was collected by centrifugation. Proteins were alkylated with iodoacetamide, purified by acetone precipitation, and subsequently dissolved in DB buffer (8 M urea, 100 mM TEAB, pH 8.5). Protein concentration was determined using the Bradford assay with a BSA standard curve (0–0.5 μg/μL), and quality was verified using SDS-PAGE (12% gel, Coomassie Brilliant Blue staining). For proteolytic digestion, proteins were subjected to tryptic hydrolysis (37 °C for 4 h, followed by overnight incubation). The reaction was terminated by acidification, and the peptides were desalted using C18 cartridges prior to lyophilization [23].

#### 2.4.2. Liquid Chromatography Tandem Mass Spectrometry (LC-MS/MS) Analysis in DIA Mode

Chromatographic separation was performed on a Vanquish Neo UHPLC system (Thermo Fisher, Bremen, Germany) equipped with a C18 guard column (0.5 × 300 mm, 5 μm) and an ES906 analytical column (150 × 15 cm, 2 μm) maintained at 50 °C. The mobile phase consisted of (A) 0.1% formic acid in water and (B) 0.1% formic acid in 80% acetonitrile. Mass spectrometric analysis was conducted using an Orbitrap Astral mass spectrometer (Thermo Fisher, Bremen, Germany) operated in data-independent acquisition (DIA) mode with the following parameters: a primary mass scan range of *m*/*z* 380–980 at a resolution of 240,000 (at *m*/*z* 200), a secondary mass scan range of *m*/*z* 150–2000 at a resolution of 80,000, normalized collision energy of 25%, and 300 predefined DIA windows [24].

#### 2.4.3. Bioinformatics Analysis of Differentially Expressed Proteins (DEPs)

Protein identification was performed against the UniProt database (Anas_platyrhynchos_uniprot_2024_07_26.fasta) using DIA-NN 1.8.1 software, with stringent quality control to retain only peptide-spectrum matches (PSMs) at >99% confidence (FDR-corrected). For quantitative analysis, precursor ion chromatograms and fragment ion matching were processed in DIA-NN, applying thresholds of Global.Q.Value < 0.01 and PG.Q.Value < 0.01. Differentially expressed proteins (DEPs) were identified by pairwise group comparisons using *t*-tests (|log2 FC| ≥ 1, *p* < 0.05). Functional annotation integrated InterProScan for GO terms and IPR domain analysis, supplemented by COG classification and KEGG pathway enrichment. Protein–protein interaction networks were predicted via STRING (v.11.5), with the results visualized through volcano plots and hierarchical clustering heatmaps.

### 2.5. Metabolomics Analysis

#### 2.5.1. Sample Extraction

Six biological replicates were randomly selected per experimental group (*n* = 6), with approximately 100 mg of tissue samples homogenized in liquid nitrogen and transferred to EP tubes. Each sample was extracted with 500 μL of 80% methanol aqueous solution, followed by vortex mixing and incubation on ice for 5 min. After centrifugation at 15,000× *g* for 20 min at 4 °C, an aliquot of the supernatant was diluted with MS-grade water to achieve a final methanol concentration of 53%. The diluted extract was recentrifuged under identical conditions (15,000× *g*, 20 min, 4 °C), and the resulting supernatant was collected for subsequent LC-MS analysis.

#### 2.5.2. High-Performance Liquid Chromatography (HPLC)–MS Analysis

Chromatographic separation was performed on a Vanquish UHPLC system (Thermo Fisher, Bremen, Germany) equipped with a Hypersil Gold C18 column, using a mobile phase consisting of 0.1% formic acid in water (A) and methanol (B). The separation was conducted at a column temperature of 40 °C with a flow rate of 0.2 mL/min. Mass spectrometric analysis was carried out using an electrospray ionization (ESI) source operated in positive/negative switching mode with the following optimized parameters: a spray voltage of 3.5 kV, sheath gas flow at 35 psi, auxiliary gas flow at 10 L/min, a capillary temperature of 320 °C, an S-lens RF level of 60, and an auxiliary gas heater temperature of 350 °C. Full-scan mass spectra were acquired over the m/z range 100–1500, followed by data-dependent acquisition for MS/MS analysis.

#### 2.5.3. Bioinformatics Analysis of Differentially Expressed Metabolites (DEMs)

Mass spectral data were converted to mzXML format (ProteoWizard) and processed using XCMS for feature extraction, retention time alignment, and QC-based normalization. Metabolites were identified (±10 ppm mass accuracy) through spectral library matching. Data were blank-subtracted, normalized to QC samples, and filtered (CV < 30% in QCs). Metabolites were annotated against KEGG/HMDB/LIPIDMAPS databases. Multivariate (PCA/PLS-DA) and univariate analyses (*t*-test; VIP > 1, *p* < 0.05, |log2 FC| ≥ 1) were performed. Visualization included volcano plots, z-score heatmaps, and correlation networks. Pathway enrichment was assessed via hypergeometric testing (KEGG; *p* < 0.05).

### 2.6. Statistical Analysis

Data were preliminarily collated and calculated using Microsoft Excel 2010. Prior to Two-way ANOVA, the normality of data distribution was verified (Shapiro–Wilk test), confirming that the data followed a normal distribution. Then, the data were subjected to Two-way ANOVA (Analysis of Variance) using SPSS 25.0 (SPSS software for Windows; SPSS Inc., Chicago, IL, USA). Means were tested for significance using the LSD method. All pairwise comparisons were performed using Tukey’s Honestly Significant Difference (HSD) test to control the error rate. The results were presented as the mean ± standard deviation (mean ± SD), and a significant difference was indicated by *p* < 0.05. The formula for ΔE, as defined by the International Commission on Illumination (CIE), was applied as follows:

$$\Delta E\;=\sqrt[{2}]{\left[{\left(\Delta L*\right)}^{2}+{\left(\Delta a*\right)}^{2}+{\left(\Delta b*\right)}^{2}\right]}$$

## 3. Results

### 3.1. Effect of Age and Muscle Part on Duck Meat Quality Characteristics

To reveal the difference in duck meat quality at different ages and in different parts, analysis of the physical properties of breast and thigh muscle was performed (Table 1). The results showed that the shear force value of the same muscle part increased significantly with the increase in age; at the same age, the shear force value of thigh muscles was significantly higher than that of breast muscles. The analysis of a* values revealed that the a* values of 468-day-old ducks were higher than those of 90-day-old ducks for the same muscle part (*p* < 0.05). However, no significant differences in pH value, b* value, and L* value were observed among these four types of muscles (*p* > 0.05). Colorimetric analysis revealed perceptible to clearly perceptible differences (ΔE = 2.08–5.35) between all compared groups. The largest color shifts (ΔE > 5.0) were observed between the D90 and D468 sample types (Table 2).

### 3.2. Identification and Comparison of DEPs at Different Ages and in Meat Parts

**Figure 1:**
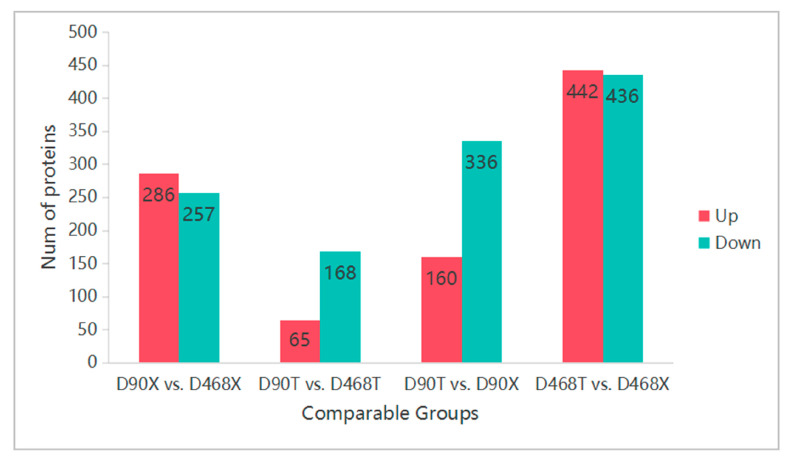
Number of DEPs in different muscles of duck meat.

The DIA combined with the UHPLC-Astral LC/MS method was used to isolate and identify proteins from the four groups. In each comparison group, those with *p* < 0.05 and FC ≥ 1.2 or FC < 0.83 were considered DEPs. The D90X vs. D468X group had 543 DEPs, the D90T vs. D468T group had 233 DEPs, the D90T vs. D90X group had 496 DEPs, and the D468T vs. D468X group had 878 DEPs. Among the DEPs, 286, 65, 160, and 442 DEPs were upregulated in the four groups, whereas 257, 168, 336, and 436 DEPs were downregulated (Figure 1).

### 3.3. Enrichment Analysis of the Characteristic DEPs

#### 3.3.1. GO Functional Classification of DEPs

The protein functional analysis was conducted using all DEPs based on the GO category enrichment and GO and UniProt databases. When comparing groups of different ages, the biological process category was involved in metabolic processes and oxidation-reduction processes; in the cellular component category, the enriched GO terms were primarily associated with intracellular organelle parts; and in the molecular function category, the DEPs were mainly related to oxidoreductase activity and peptidase activity (Figure S1A,B). For comparisons between two muscle groups, the DEPs in the biological process category were involved in oxidation–reduction processes and single-organism processes; in the cellular component category, the enriched GO terms were mainly involved in intracellular organelle parts, mitochondrial parts, plasma membranes, and membranes; and in the molecular function category, the DEPs were primarily related to oxidoreductase activity (Figure S1C,D).

#### 3.3.2. KEGG Pathway Analysis of DEPs

**Figure 2:**
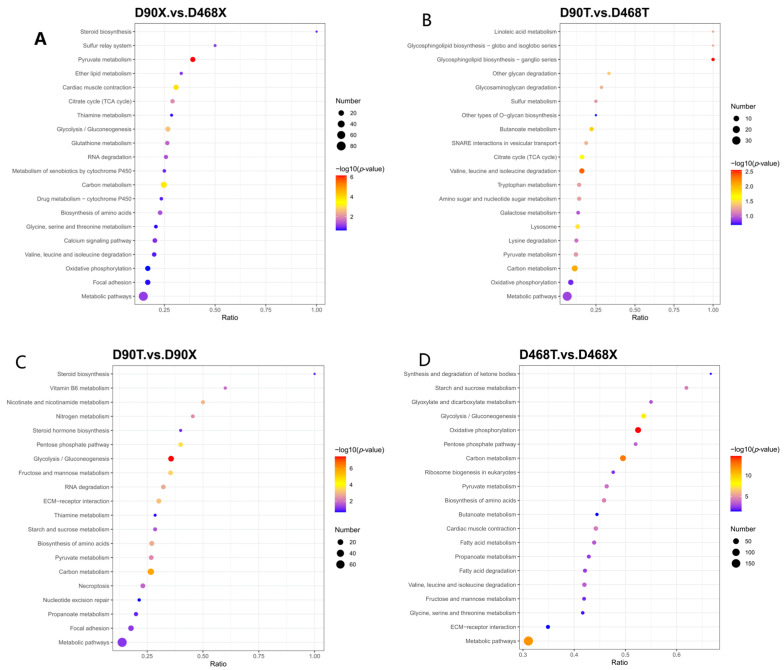
Bubble diagram of KEGG metabolic pathway enrichment analysis of DEPs. (**A**) D90X vs. D468X; (**B**) D90T vs. D468T; (**C**) D90X vs. D90T; (**D**) D468X vs. D468T.

As shown in Figure 2 the pathways of differential proteins in different age groups were found to differ significantly, with the focus being on oxidative phosphorylation and carbon metabolism. The key pathways of glucose metabolism included pyruvate metabolism, glycolysis, and other pathways. Fat and ketone body metabolism included fatty acid metabolism, ketone body synthesis, and degradation pathways. Finally, anabolic metabolism included amino acid biosynthesis, nucleotide metabolism, and other pathways. With regard to muscle site specificity, the pathways of the different proteins exhibited significant disparities, primarily within the ribosome biogenesis pathway, RNA degradation pathway, and ‘myocardial contraction’ pathway.

#### 3.3.3. Protein–Protein Interaction Analysis

Protein–protein interaction (PPI) networks were further established for DEPs by referring to the STRING database. In the D90X vs. D468X comparison group, the differential protein PPI network contained 37 nodes and 129 linkages, namely 37 proteins and 129 interactions (Figure S2A). Of these, cytochrome c oxidase subunit 4 (ROK1N1) demonstrated the strongest interaction with six proteins. In the D90T vs. D468T comparison group, the differential protein PPI network comprised 48 proteins and 109 interactions (Figure S2B). Among them, MRPL38 (A0A493U2X3) and MRPS9 (U3J2L4) exhibited the strongest interactions with six proteins. In the D90T vs. D90X comparison group, the differential protein PPI network comprised 62 proteins and 124 interacting relationships (Figure S2C). Among them, GPI (A0A8B9SJ62) and PKM (A0A493SUY8) demonstrated the strongest interactions with six proteins. In the D468T vs. D468X comparison group, the differential protein PPI network comprised 31 proteins and 115 interactions (Figure S2D). Of these, NDUFV2 (A0A8B9TE39) and NDUFS3 (A0A493SXF5) demonstrated the strongest interactions with 12 proteins.

### 3.4. Analysis of Correlation Between Meat Quality Indicators and DEPs

#### 3.4.1. Analysis of the Correlation Between Meat Quality and DEPs in Different Muscle Parts of 468-Day-Old Sansui Ducks

Differences in pectoral and thigh muscle quality characteristics were selected for correlation analysis with DEPs in 468-day-old Sansui ducks (Table 3). Pearson’s method was used to analyze the correlation between the relative content values of DEPs and shear force, pH, brightness (L*), redness (a*), and yellowness (b*) in D468T and D468X groups. The 23 DEPs were screened to obtain highly significant correlations (*p* < 0.01) with the shear force of Sansui duck meat, of which b* was significantly correlated only with A0A493TGA7 (r = 0.821; *p* < 0.05), and A0A8B9STW6, A0A493TM94, R0LG97, and R0LPZ6 were significantly correlated with pH (r = −0.93, −0.841, −0.906 and 0.822; *p* < 0.05). One DEP A0A8B9TGK7 (r = 0.889) was significantly correlated with only L*.

#### 3.4.2. Analysis of the Expression Levels and Relative Quantitative Value of Significantly Correlated DEPs on Different Muscle Parts

As shown in Table 4, 28 DEPs with significant correlations with Sansui duck meat quality indices exhibited significant differences in relative quantification across different muscle parts (*p* < 0.05). Specifically, the relative levels of A0A8B9STW6 (Neuralized E3 ubiquitin protein ligase 2), A0A493TM94 (Heterogeneous nuclear ribonucleoprotein A3), and R0LG97 (Dystrophin) were significantly higher in Group T than in Group X (*p* < 0.05), with all three showing negative correlations with pH. Additionally, A0A8B9TGK7 (Peptidyl-prolyl cis-trans isomerase) displayed a significantly higher relative quantification in Group T compared to Group X (*p* < 0.05) and a significant positive correlation with brightness. Conversely, the relative level of A0A493TGA7 in Group T was significantly lower than that in Group X (*p* < 0.05), while exhibiting a significant positive correlation with yellowness.

#### 3.4.3. Analysis of the Correlation Between Breast Meat Quality and DEPs in Sansui Ducks at Different Ages

Using Pearson’s correlation method, the relative contents of differential proteins associated with shear force, pH, brightness (L*), redness (a*), and yellowness (b*) were analyzed in the D90X and D468X groups, with the results presented in Table 5. These results indicate that meat quality traits (shear force, pH, b*, and L*) were primarily influenced by metabolic enzymes, showing significant correlations mainly with the differential proteins A0A493TG46, A0A493TIN0, A0A493TJU7, A0A493TWK1, and A0A8B9T8F1.

#### 3.4.4. Analysis of the Expression Levels and Relative Quantitative Value of Significantly Correlated DEPs in Different Age Groups

As shown in Table 6, the relative quantitative values of 18 differential proteins significantly correlated with meat quality indexes of Sansui ducks were significantly (*p* < 0.05) different at different ages, and these significant differential proteins might be the key influencing proteins contributing to the meat quality characteristics of the 90 days of age group versus the 468 days of age group. The relative quantitative values of proteins A0A493TG46, A0A493TIN0, and A0A493TJU7 were significantly upregulated (*p* < 0.05) and the relative quantitative values of proteins A0A493TWK1, A0A8B9SHF6, and R0JG91 were significantly downregulated (*p* < 0.05) with the increase in days of age.

### 3.5. Metabolic Profiling Analysis of Characteristic Metabolites at Different Ages and in Meat Parts

#### 3.5.1. Identification of Metabolites

**Figure 3:**
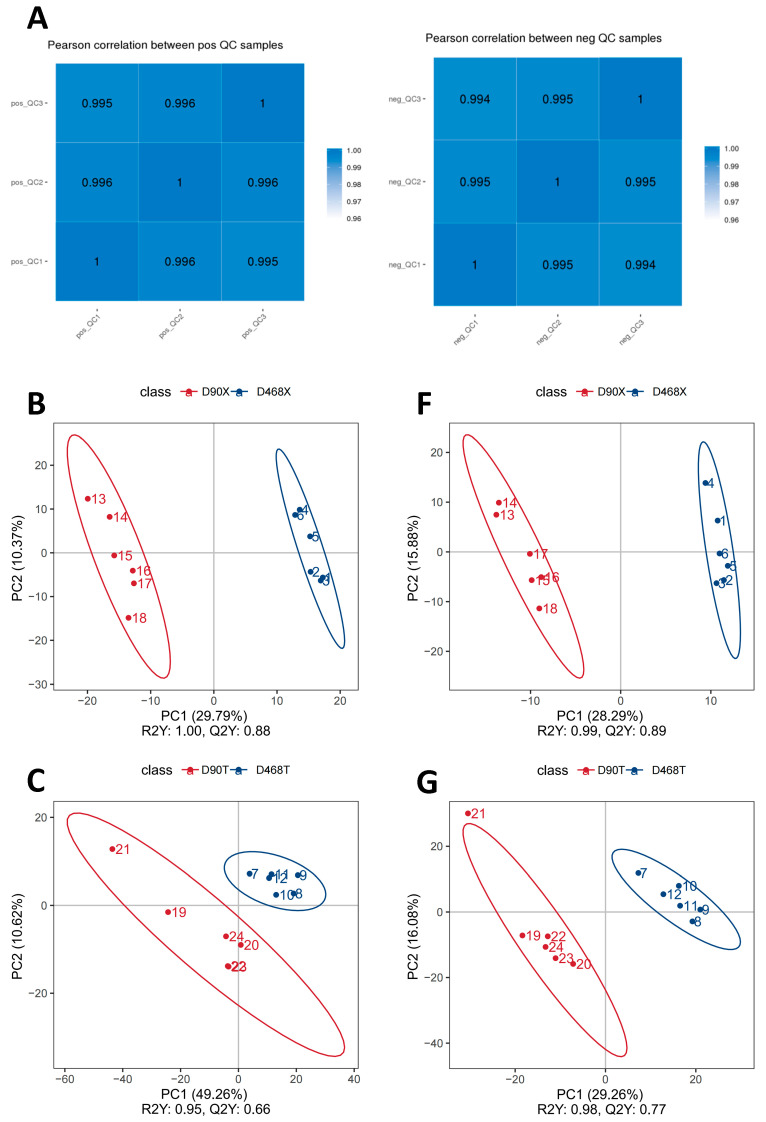

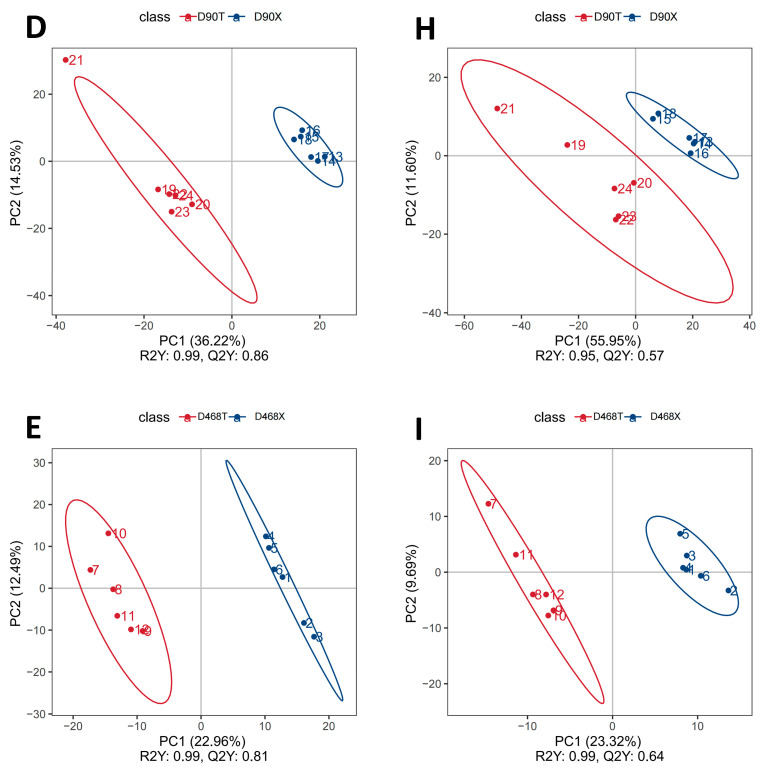
Combined analysis of QC sample correlation analysis plot and PLS-DA score plot. (**A**) QC sample correlation analysis plot. (**B**) D90X vs. D468X in positive mode; (**C**) D90T vs. D468T in positive mode; (**D**) D90X vs. D90T in positive mode; (**E**) D468X vs. D468T in positive mode; (**F**) D90X vs. D468X in negative mode; (**G**) D90T vs. D468T in negative mode; (**H**) D90X vs. D90T in negative mode; (**I**) D468X vs. D468T in negative mode.

Differential metabolite variability in Sansui duck meat quality at different days of age and in different muscle parts was investigated via untargeted metabolomics analysis using UHPLC-MS/MS technology. Combining the analysis based on the QC sample correlation analysis plot (Figure 3A) and PLS-DA score plot (Figure 3B–I), we observe that the metabolome data measured in the present study exhibit good repeatability and reliability.

#### 3.5.2. Screening and Analysis of DEMs

The DEMs were selected based on the OPLS-DA model criteria of VIP > 1, FC > 1.5 or FC < 0.667 and *p* < 0.05. A comprehensive overview of the number of DEMs identified in both positive and negative ionization modes for all pairwise comparisons is presented in Table 7. In general, the comparison between anatomic locations in the D90T vs. D90X group yielded the highest number of DEMs, particularly in positive mode. Furthermore, the D90X vs. D468X group showed predominant upregulation of metabolites, whereas the D90T vs. D468T group was characterized by predominant downregulation.

#### 3.5.3. KEGG Pathway Classification Analysis of DEMs

**Figure 4:**
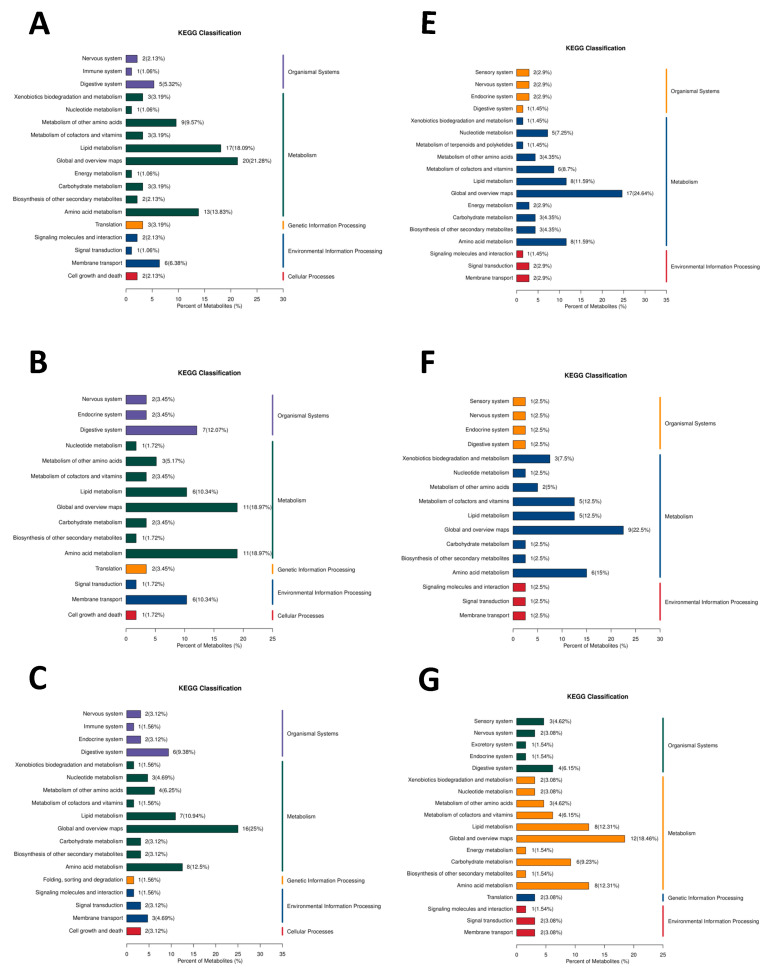

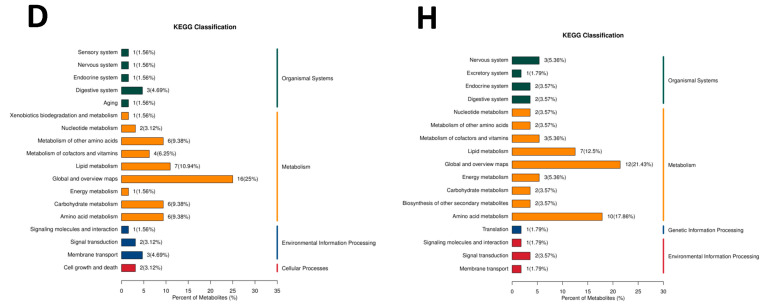
Metabolic KEGG classification by DEMs. (**A**) D90X vs. D468X in positive mode; (**B**) D90T vs. D468T in positive mode; (**C**) D90X vs. D90T in positive mode; (**D**) D468X vs. D468T in positive mode; (**E**) D90X vs. D468X in negative mode; (**F**) D90T vs. D468T in negative mode; (**G**) D90X vs. D90T in negative mode; (**H**) D468X vs. D468T in negative mode.

Furthermore, KEGG pathway classification analysis reveals that there were 96, 58, 64, and 64 differential metabolites annotated to pathways in the D90X vs. D468X, D90T vs. D468T, D90T vs. D90X, and D468T vs. D468X groups, respectively, in positive mode, as shown in Figure 4A–D. In the D90X vs. D468X and D90T vs. D468T comparison groups, the DEMs mainly annotated to the metabolic pathways of lipid metabolism, amino acid metabolism, and the digestive system. Similarly, in the D90T vs. D90X and D468T vs. D468X comparison groups, the DEMs were mainly annotated to amino acid metabolism, lipid metabolism, and digestive system pathways. DEMs in the D90X vs. D468X, D90T vs. D468T, D90T vs. D90X, and D468T vs. D468X groups in negative mode were annotated with 69, 40, 65, and 56 pathway-annotated metabolites, respectively, as shown in Figure 4E–H. In the D90X vs. D468X and D90T vs. D468T comparative groups, the DEMs mainly annotated to the metabolic pathways of lipid metabolism and amino acid metabolism. In the D90T vs. D90X and D468T vs. D468X comparison groups, the DEMs were mainly annotated to amino acid metabolism, lipid metabolism, and energy metabolism.

#### 3.5.4. KEGG Pathway Enrichment Analysis of DEMs

**Figure 5:**
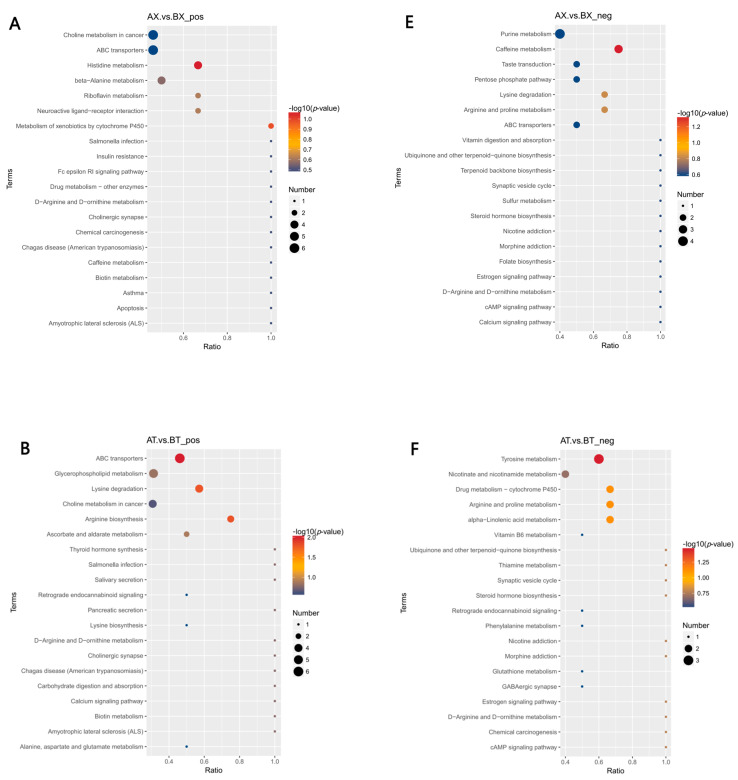

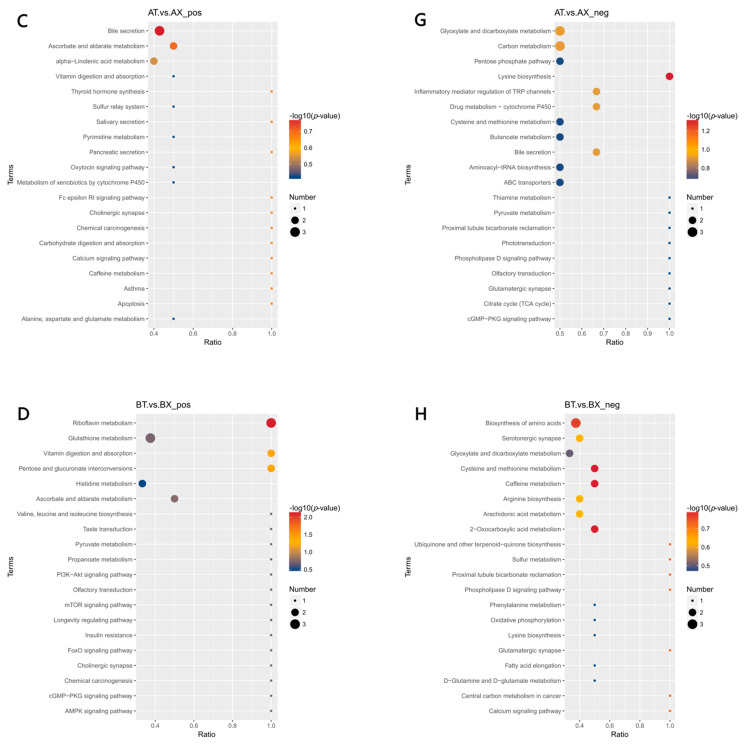
An enrichment map of the top 20 metabolic pathways. (**A**) D90X vs. D468X in positive mode; (**B**) D90T vs. D468T in positive mode; (**C**) D90X vs. D90T in positive mode; (**D**) D468X vs. D468T in positive mode; (**E**) D90X vs. D468X in negative mode; (**F**) D90T vs. D468T in negative mode; (**G**) D90X vs. D90T in negative mode; (**H**) D468X vs. D468T in negative mode.

The differential metabolites were submitted to the KEGG website for enrichment analysis of the relevant pathways. The top 20 most significant pathways were selected to plot the KEGG enrichment scatterplot. As shown in Figure 5, in positive mode, the main metabolic pathways with significant differences in KEGG enrichment in the D90X vs. D468X group included histidine metabolism and metabolism of xenobiotics by cytochrome P450, of which histidine metabolism had the greatest effect on this group. The main metabolic pathways involved in KEGG enrichment in the D90T vs. D468T group were ABC transporters, lysine degradation, and arginine biosynthesis. The main metabolic pathways involved in KEGG enrichment in the D90T vs. D90X group were bile secretion, ascorbate and aldarate metabolism, and the metabolism of xenobiotics by cytochrome P450. Riboflavin metabolism was the most significant and highly enriched in the D468T vs. D468X group. In negative mode, in the D90X vs. D468X group, caffeine metabolism was the most significantly enriched pathway among the differentially abundant metabolites. For the D90T vs. D468T group, tyrosine metabolism exhibited the highest enrichment significance in differential metabolites.

In the D90T vs. D90X group, pathways such as lysine biosynthesis, glyoxylate and dicarboxylate metabolism, carbon metabolism, inflammatory mediator regulation of TRP channels, drug metabolism—cytochrome P450, and bile secretion showed moderate enrichment significance. Between D468T and D468X, the most significantly enriched differential metabolites were associated with the biosynthesis of amino acids, cysteine and methionine metabolism, caffeine metabolism, and 2-oxocarboxylic acid metabolism.

### 3.6. Analysis of Correlation Between Meat Quality Indicators and DEMs

#### 3.6.1. Analysis of the Correlation Between Meat Quality and DEMs in Different Muscle Parts of 468-Day-Old Sansui Ducks

Pearson correlation analysis was conducted to investigate the associations between these DEMs and meat quality parameters in 468-day-old Sansui ducks, with a specific focus on metabolites significantly correlated with shear force. The results demonstrated that multiple dipeptides and oligopeptides exhibited consistent negative correlations with shear force value, as shown in Table 8. Notably, histidylmethionine (r = −0.60, *p* < 0.05), hemorphin-4 (r = −0.66, *p* < 0.01), and L-valyl-L-phenylalanine (r = −0.64, *p* < 0.05) showed strong inverse relationships with shear force, suggesting their potential role in modulating meat tenderness through proteolytic regulation or protein–protein interactions, given their downregulation in pectoral muscle (X) compared to thigh muscle (T).

Of particular interest is N-decanoylglycine, which displayed a highly significant positive correlation with pH (r = 0.84, *p* < 0.01) and a moderate positive association with shear force (r = 0.54, *p* < 0.05), indicating its potential as a biomarker for postmortem glycolytic activity and subsequent texture development. Additionally, (6Z,9Z)-hexadecadienoylcarnitine exhibited a strong negative correlation with lightness (L*) (r = −0.68, *p* < 0.01), implying its involvement in myoglobin redox status and meat color formation.

#### 3.6.2. Analysis of the Correlation Between Breast Meat Quality and DEMs in Sansui Ducks at Different Ages

Pearson correlation analysis revealed significant associations between these DEMs and quality traits in breast meat of Sansui ducks at different developmental stages (90 days vs. 468 days). (2R,4R)-4-aminopyrrolidine-2,4-dicarboxylic acid demonstrated particularly strong positive correlations with both pH (r = 0.92, *p* < 0.01) and shear force (r = 0.60, *p* < 0.05), while showing a negative correlation with L* (r = −0.55, *p* < 0.05), suggesting its potential role in postmortem biochemical processes affecting meat texture and color. Several metabolites including 4-keto-clonostachydiol (r = 0.90, *p* < 0.01 for pH; r = 0.76, *p* < 0.01 for shear force) and 2-hydroxyindecanoic acid (r = 0.90, *p* < 0.01 for pH; r = 0.65, *p* < 0.05 for shear force) exhibited similar patterns, indicating a cluster of age-dependent metabolites influencing meat quality through pH-related mechanisms. In contrast, beta-alanyl-L-arginine showed inverse relationships with pH (r = −0.73, *p* < 0.01) and shear force (r = −0.55, *p* < 0.05), along with a positive correlation with L* (r = 0.55, *p* < 0.05), representing a distinct metabolic pathway potentially beneficial for meat quality. The phospholipid PC(18:1(11Z)/18:1(12Z)-20H(9,10)) followed a comparable pattern (r = −0.69, *p* < 0.01 for P H; r = −0.54, *p* < 0.05 for shear force), highlighting the importance of lipid metabolism in meat quality development, as shown in Table 9.

### 3.7. Integrative Proteomics and Metabolomics Analysis

Integrative metabolomics and proteomics analysis of Sansui duck muscle was performed to identify unique regulatory mechanisms. Based on comparative analyses of the 90X vs. 468X and 90T vs. 468T groups, co-enriched pathways containing unique metabolites and proteins were identified, with three shared signaling pathways enriched (Table 10). Based on comparative analyses of the 90X vs. 90T and 468X vs. 468T groups, co-enriched pathways containing unique metabolites and proteins were identified, with one shared signaling pathway enriched (Table 11).

## 4. Discussion

With increasing attention paid to the meat quality and nutritional value of livestock and poultry products, breeding for superior meat traits has become a key objective. This study investigated the muscle characteristics of Sansui ducks at two different ages (90 and 468 days) and from two muscle types (breast and thigh muscles). Through integrated proteomics and metabolomics analysis, it identified proteins and key metabolites contributing to meat quality differences, thereby laying a foundation for elucidating the molecular mechanisms underlying meat quality formation in Sansui ducks.

Shear force is a core indicator of meat tenderness. The results showed that shear force increased with age and was significantly higher in thigh muscles than in breast muscles. This finding is consistent with the general characteristics of avian muscle development: as age increases, connective tissue cross-linking—particularly collagen—intensifies, and muscle fiber diameter thickens, leading to tougher muscle texture and elevated shear force [21,25,26]. The variation between muscle types stems from their functional differentiation. Thigh muscles, which support prolonged locomotion, are dominated by slow-twitch fibers (red muscle), characterized by a well-developed sarcoplasmic reticulum, denser muscle fiber arrangement, and higher collagen content compared to breast muscles. In contrast, breast muscles are primarily composed of fast-twitch fibers (white muscle), which are thicker but more loosely arranged, resulting in lower shear force [27,28].

Regarding meat color indicators, the a* value (redness) of 468-day-old ducks was significantly higher than that of 90-day-old ducks, indicating that muscle redness deepens with age. The a* value is mainly determined by the content and oxidation state of myoglobin. Older animals tend to accumulate more myoglobin, and the proportion of slow-twitch fibers may be higher—especially in thigh muscles. Since slow-twitch fibers contain substantially more myoglobin than fast-twitch fibers, this results in a higher redness value [29,30,31]. In summary, differences in the physical properties of Sansui duck muscle are primarily reflected in pH and a* value, with synergistic regulation by age and muscle type. The core mechanisms are likely related to muscle fiber type composition, glycogen metabolism, and myoglobin accumulation. While muscle fiber type differences affect functional properties like shear force and redness, they may not necessarily translate to divergent color characteristics in terms of lightness and yellowness in this specific breed.

Unlike shear force and a*, pH, b*, and L* showed no significant differences across age groups or muscle regions, reflecting the metabolic stability of Sansui duck muscle. pH is primarily influenced by the amount of lactic acid produced through postmortem glycolysis; its stability suggests minimal variation in glycolytic rates among muscles of different ages and regions, potentially due to breed-specific regulatory mechanisms of glucose metabolism in Sansui ducks [32]. Yellowness (b*) is typically associated with carotenoid content and the degree of lipid oxidation; its consistency indicates that the accumulation of fat-soluble pigments and the extent of lipid oxidation are less affected by age or muscle type [33]. Lightness (L*), which correlates with muscle water distribution and myofibrillar density, also exhibited no significant variation, likely due to the relatively uniform water-holding capacity and muscle fiber compactness across regions, which remain stable with age or functional differentiation [34]. The similar L* and b* values observed between breast and thigh muscles, despite their different fiber type composition, reflect the complex interplay of multiple factors influencing meat color. Similar results were observed in studies of Bactrian camels, where no differences were found in the yellowness and brightness of meat color across the semitendinosus, psoas major, and longissimus dorsi muscle sections [35]. Furthermore, considering the potential color gradient across different muscle depths, we shall incorporate a chromaticity measurement protocol for meat color in future studies. This will include additional measurement points at distinct depths (superficial, intermediate, and deep layers) to capture existing color gradients and provide chromaticity distribution maps with enhanced spatial resolution.

To further investigate the mechanisms underlying meat quality differences across age groups and muscle regions, proteomics analysis was conducted. Differentially expressed protein (DEP) profiles revealed that the number of DEPs between muscle types at the same age was substantially greater than that between age groups within the same muscle. This pattern reflects functional differentiation: breast muscles, composed primarily of fast-twitch fibers, are adapted for rapid, explosive movements and rely heavily on glycolysis for energy, resulting in more dynamic protein expression and metabolic activity [36]. In contrast, thigh muscles, composed of slow-twitch fibers, support sustained movement and primarily utilize oxidative metabolism, exhibiting more stable structural protein expression and fewer molecular changes. This expression pattern suggests that protein-level adjustments are driven more by long-term physiological demands—such as the structural requirements of continuous muscle activity—than by age-related or short-term environmental factors. A previous study in chickens identified 322 DEPs between breast and thigh muscles, with 129 highly expressed in breast muscles and 193 in thigh muscles [37].

Functional enrichment analysis revealed that differentially expressed proteins (DEPs) in the proteome influence meat quality primarily by regulating core biological pathways. Energy metabolism pathways serve as key regulatory nodes: DEPs involved in pathways such as oxidative phosphorylation and glycolysis collectively reshape muscle energy supply modes. In the breast muscles of 468-day-old ducks, upregulation of oxidative phosphorylation-related proteins (e.g., cytochrome c oxidase subunit 4) was accompanied by downregulation of glycolysis-related proteins, indicating a shift in energy metabolism from glycolysis-dominant to oxidative metabolism-dominant. This transition likely contributed to elevated pH levels, as reduced glycolytic activity leads to lower lactic acid production [38]. Previous studies have shown that key glycolytic enzymes—such as lactate dehydrogenase B (LDHB) and enolase 1 (ENO1)—also exhibit differential expression in muscle tissues, reflecting adaptations to distinct metabolic states [39]. Additionally, the ribosome biogenesis pathway was enriched among DEPs in thigh muscles, suggesting a high demand for structural protein synthesis to maintain myofibrillar integrity during sustained movement. Enrichment of DEPs associated with peptidase activity in age-specific groups further highlights the critical role of proteolysis in muscle maturation, particularly in regulating myofibrillar protein degradation and its impact on tenderness [40,41]. Similar findings were reported by Van et al. (2007), who reported age-related differences in protease and lipase activity during muscle development in pigs [42].

Correlation analysis indicated that DEPs directly influence phenotypes through structural stabilization and enzymatic activity regulation. Among 23 DEPs strongly correlated with shear force, dystrophin—a sarcolemma-stabilizing protein—was highly expressed in thigh muscles, contributing to increased shear force by enhancing myofibrillar structural integrity [15,43]. Similarly, Zhang et al. (2018) identified structural proteins and metabolic enzymes through proteomics analysis. The expression levels of these proteins were found to correlate significantly with muscle water retention capacity [13]. A0A8B9STW6, an E3 ubiquitin ligase also highly expressed in thigh muscles, may indirectly elevate pH by targeting glycolytic enzymes for degradation, thereby inhibiting postmortem glycolysis. With increasing age, upregulation of oxidative phosphorylation-related proteins (e.g., NDUFV2) may contribute to increased shear force in older ducks by suppressing glycolysis and promoting collagen cross-linking—consistent with the observed phenotype of age-related meat toughening [44,45]. Furthermore, glutathione S-transferase (GST, A0A8B9SHF6) was downregulated in the breast muscles of 468-day-old ducks, potentially impairing antioxidant defense via the Nrf2/ARE pathway. Malheiros et al. (2021) similarly identified proteins such as antioxidant proteins in their proteomics study of Nellore bulls, which were found to be closely associated with meat tenderness characteristics [46]. This may lead to the accumulation of lipid peroxidation products such as malondialdehyde (MDA), which is associated with a significant increase in muscle redness (a* value). This observation aligns with findings by Liu et al. (2022) regarding beef water-holding capacity. Similarly, Wang et al. (2018) reported that MDA promotes metmyoglobin formation in rabbit meat, resulting in meat discoloration [47,48]. These results collectively suggest that the proteome functions as a core regulatory hub for meat quality differences, primarily through the modulation of structural stability and metabolic pathway activity.

Subsequently, the mechanisms underlying meat quality differences in Sansui ducks were further explored using metabolomics analysis. The distribution of differential metabolites (DEMs) exhibited a pattern broadly consistent with that of proteomics, though with subtle distinctions. Specifically, the number of DEMs between different muscle regions at the same age was more prominent, and the number of DEMs identified in breast muscles across age groups was slightly higher than that observed in the proteome. This difference is attributed to the nature of metabolites as intermediates in metabolic pathways, which allows them to respond more rapidly to environmental and physiological changes, thereby capturing short-term physiological fluctuations with greater sensitivity [49].

Functional enrichment analysis revealed that DEMs were primarily enriched in downstream pathways such as amino acid metabolism and lipid metabolism, functioning as “functional executors” of protein-regulated pathways. DEMs involved in amino acid metabolism (e.g., histidine metabolism intermediates) contribute to the generation of umami precursors, directly impacting flavor quality. DEMs associated with lipid metabolism (e.g., metabolites involved in bile secretion) enhance lipid absorption in thigh muscles, supporting their elevated oxidative metabolic activity [50]. In metabolomics studies of beef, such metabolites have been shown to indirectly influence energy supply and metabolic adaptability in muscle tissue by modulating fat digestion and absorption [51]. These metabolic pathways form a hierarchical regulatory structure with the proteome, characterized by an “upstream regulation–downstream execution” relationship. For instance, the proteome-driven shift toward oxidative phosphorylation is reflected at the metabolic level by reduced concentrations of glycolysis-related metabolites such as pyruvate.

In relation to meat quality traits, DEMs in the metabolome influence key phenotypes through direct material action, forming a synergistic regulatory network with the proteome. In the regulation of shear force, dipeptides such as histidine–methionine were highly abundant in breast muscles, where they likely reduce shear force by promoting myofibrillar protein hydrolysis. This effect complements the proteomics finding of upregulated DEPs involved in ribosome biogenesis in thigh muscles, which enhance structural protein synthesis. Together, these factors contribute to the higher shear force observed in thigh muscles compared to breast muscles [52,53]. Previous metabolomics studies have shown that meat flavor is influenced by various metabolites, including amino acids, fatty acids, and volatile organic compounds. Moreover, reduced levels of glycolytic intermediates may affect the synthesis of these flavor-related substances, thereby altering meat flavor profiles [54]. Similarly, Shi Y et al. (2019) utilized metabolomics to reveal that the flavor formation of ham is primarily determined by volatile constituents, which are predominantly composed of aldehyde and alcohol compounds [55]. In terms of pH regulation, *N*-decanoylglycine exhibited a strong positive correlation with pH (r = 0.84), suggesting that it slows postmortem pH decline by inhibiting glycolytic enzyme activity. This action may interact with the proteomics regulator A0A8B9STW6, producing an additive effect on pH stabilization [56,57]. With advancing age, metabolites such as 4-ketoclostestosterone accumulated in the muscle tissue, acting in concert with the upregulation of oxidative phosphorylation proteins in the proteome. This synergy likely contributes to increased shear force by suppressing glycolysis and promoting collagen cross-linking. Similarly, Gu et al. (2024) observed that meat from older ducks exhibited greater firmness [8]. Metabolomics analysis further revealed reduced levels of guanosine, hypoxanthine, guanine, and doxepin in the meat of older ducks, indicating enhanced nutritional value. Furthermore, Subbaraj et al. (2016) utilized metabolomics to uncover the association mechanism between polar metabolites in mutton and myoglobin stability, confirming a positive correlation between antioxidant content and meat color stability [18]. It is worth noting that significant differences in meat quality between breeds were also revealed through metabolomics analysis. Jung et al. (2010) identified succinic acid and characteristic amino acids such as isoleucine and leucine as key metabolites distinguishing beef from different origins, including Australia and the United States [58]. Wang et al. (2017) compared the metabolomics differences between Peking ducks and Linwu ducks, revealing significant breed-specificity in metabolites such as carnosic acid derivatives and succinate [59]. It is worth mentioning that metabolites serve as immediate substrates and products of enzymatic reactions, enabling rapid response to physiological changes within minutes to hours, potentially contributing to the observed individual variability in meat quality traits. Collectively, these findings suggest that the metabolome, through the accumulation and functional activity of key metabolites, plays a pivotal role in shaping meat quality traits via interactions with proteomics pathways.

Integrated analysis facilitates the dissection of complex mechanisms underlying animal physiology and production performance. For instance, in studies on camel meat, oxidative phosphorylation, the tricarboxylic acid (TCA) cycle, and glycolysis were highlighted as key discriminatory pathways between distinct muscle types [36,60]. Additionally, Gu et al. (2024) identified the purine metabolism pathway as a key factor potentially influencing the meat quality of aged ducks through the integration of proteomics and metabolomics [20]. This study integrated proteomics–metabolomics analysis and identified three age-related co-enriched pathways (lysine degradation, butyrate metabolism, and 2-oxocarboxylic acid metabolism) and one region-related co-enriched pathway (ABC transporters), reflecting functional coupling between proteins and metabolites. In the Lysine degradation pathway: upregulation of A0A8B9TTI1 (Lysine dehydrogenase) in the proteome synergizes with the accumulation of lysine intermediates in the metabolome. This synergy enhances flavor by generating glutamic acid (an umami amino acid), consistent with the phenotypic increase in umami intensity in 468-day-old ducks [61,62]. In the ABC transporter pathway, R0JXJ3 (MRP1) in the proteome associates with choline and L-glutamine in the metabolome. MRP1 participates in phospholipid synthesis (affecting fat distribution) via choline transport and promotes the accumulation of L-glutamine (an umami precursor), explaining the molecular basis for the superior flavor of thigh muscles relative to pectoral muscles [63].

In summary, significant differences were observed in the proteomics and metabolomics profiles related to meat quality in Sansui ducks (*Anas platyrhynchos domestica*) between two muscle types and developmental stages. These differences result from the interplay of gene regulation, nutrient metabolism, energy production, and intracellular homeostasis. The integrated proteomics–metabolomics analysis identified potential biomarkers—including R0JXJ3 (MRP1), choline, and L-glutamine—that clarify the molecular mechanisms of meat quality formation. These biomarkers offer promising targets for the precise evaluation and regulation of meat quality in poultry. It should be specifically noted that this study employed only female Sansui ducks. Whilst this design enhances the internal validity of comparisons between different ages and muscle types, it simultaneously limits the generalizability of the findings to male ducks. Future research incorporating samples from both sexes may provide a more comprehensive elucidation of the specific molecular mechanisms underlying the meat characteristics of this breed. This study provides an initial exploration of the proteinome and metabolome profiles of Sansui duck meat. Subsequent validation of the proposed biomarkers may be conducted on a larger independent sample cohort using targeted mass spectrometry techniques. These biomarkers could also be integrated into genomic selection platforms. Furthermore, the meat processing industry may utilize these biomarkers for rapid, non-destructive quality assessment. Finally, quantifying these biomarkers across broader populations will assess their stability. While this study focused on different ages and anatomical variations, we acknowledge that environmental and nutritional factors can significantly modulate muscle proteome and metabolome. Dietary composition, particularly protein and lipid sources, can influence muscle metabolism and meat quality traits. Housing conditions, including stocking density and environmental enrichment, may affect stress levels and consequently alter metabolic pathways. Future studies should incorporate controlled dietary interventions and environmental monitoring to dissect these effects more precisely. Furthermore, incorporating additional time points in longitudinal studies holds significant value for establishing detailed trajectories of proteomics and metabolomics changes throughout the complete growth cycle of Sansui duck. Such investigations would provide valuable insights for optimizing production conditions to enhance meat quality.

## 5. Conclusions

This study demonstrated significant variations in meat quality attributes across different muscle types in Sansui ducks. Through integrated proteomics and metabolomics analysis, we identified key proteins, metabolites, and metabolic pathways implicated in these quality differences. Specifically, lysine degradation, butanoate metabolism, and 2-oxocarboxylic acid metabolism were discerned as central pathways contributing to age-related meat quality variations, whereas the ABC transporter pathway was closely associated with differences attributable to anatomical location. The proteins and metabolites within these pathways represent potential regulatory targets underlying the mechanisms of meat quality formation in Sansui ducks. Our findings provide valuable molecular insights into the biochemical determinants of meat quality and offer important implications for the production of high-quality poultry meat. Future studies should focus on functional validation of the identified candidate proteins and metabolites to establish causative relationships between pathway activity and meat quality traits.

## Figures and Tables

**Table 1:** Meat quality measurement results of different groups from Sansui ducks.

Measurement	Slaughter Age (d)	Muscle	
		Breast (X)	Thigh (T)
Ultimate pH	90	5.53 ± 0.21	5.96 ± 0.47
	468	5.74 ± 0.36	5.88 ± 0.56
Shear value (N)	90	7.02 ± 2.53 by	7.87 ± 3.99 bx
	468	16.99 ± 6.27 ay	32.23 ± 9.15 ax
Color parameters			
a* (redness)	90	16.31 ± 3.17 b	15.10 ± 2.16 b
	468	19.81 ± 1.63 a	19.06 ± 2.28 a
b* (yellowness)	90	9.88 ± 1.17	8.99 ± 0.92
	468	9.94 ± 0.80	9.99 ± 1.92
L* (lightness)	90	58.27 ± 3.83	55.13 ± 2.98
	468	54.22 ± 4.94	56.16 ± 4.22

x,y: means ± SD within rows with different superscript letters indicate significant differences (*p* < 0.05); a,b: means ± SD within columns between same groups with different superscript letters indicate significant differences (*p* < 0.05).

**Table 2:** Color parameter results of different groups from Sansui duck meat.

Comparison	Δa*	Δb*	ΔL*	ΔE Value	Visual Perception
D90X vs. D90T	1.209	0.885	3.145	3.48	Perceptible
D468X vs. D468T	0.754	0.048	1.939	2.08	Perceptible
D90X vs. D468X	3.496	0.063	4.055	5.35	Clearly perceptible
D90T vs. D468T	3.951	0.996	1.029	4.20	Clearly perceptible

**Table 3:** Pearson’s correlation between DEPs and quality traits of Sansui duck meat in different muscles.

Protein	Description	a*	b*	L*	pH	Shear Force
A0A493T986	Nidogen 2	−0.391	0.518	−0.247	−0.402	0.919 **
A0A8B9SIU4	Mitochondrial ribosomal protein L12	0.296	−0.641	0.390	0.176	−0.934 **
A0A8B9STW6	Neuralized E3 ubiquitin protein ligase 2	−0.234	0.084	−0.622	−0.932 *	0.419
A0A8B9TGK7	Peptidyl-prolyl cis-trans isomerase	0.601	−0.631	0.889 *	0.562	−0.664
A0A8B9TXS4	Junction plakoglobin	0.523	−0.644	0.313	0.286	−0.972 **
P19140	Alpha-enolase	−0.583	0.784	−0.494	−0.176	0.981 **
R0KF46	Collagen alpha-1(IV) chain (Fragment)	−0.384	0.588	−0.470	−0.443	0.922 **
R0LNL3	Alpha-aspartyl dipeptidase (Fragment)	0.484	−0.672	0.374	0.307	−0.973 **
A0A493SUY8	Pyruvate kinase	−0.628	0.663	−0.428	−0.379	0.938 **
A0A493SY37	Serine/threonine-protein phosphatase	−0.394	0.675	−0.396	−0.285	0.95 **
A0A493SYB4	Coiled-coil domain containing 88A	−0.522	0.704	−0.256	−0.181	0.956 **
A0A493SYW9	Fructose-1,6-bisphosphatase isozyme 2	−0.626	0.700	−0.488	−0.366	0.954 **
A0A493T2Z7	Microtubule-associated monooxygenase, calponin and LIM domain containing 2	−0.672	0.732	−0.488	−0.307	0.957 **
A0A493TGA7	Leucine-rich repeat containing 19	−0.543	0.821 *	−0.446	−0.115	0.985 **
A0A493TM94	Heterogeneous nuclear ribonucleoprotein A3	−0.472	0.316	−0.686	−0.841 *	0.579
A0A493U353	Chondroitin sulfate proteoglycan 4	−0.604	0.638	−0.493	−0.363	0.924 **
A0A8B9QQD8	Tetraspanin	−0.609	0.756	−0.567	−0.351	0.937 **
A0A8B9SJ62	Glucose-6-phosphate isomerase	−0.601	0.623	−0.405	−0.417	0.922 **
A0A8B9TJ91	Myosin binding protein H	−0.437	0.585	−0.491	−0.478	0.921 **
A0A8B9TP15	VAMP-associated protein B and C	0.429	−0.636	0.313	0.308	−0.950 **
R0KUY7	Microtubule-associated protein (Fragment)	0.583	−0.755	0.528	0.316	−0.960 **
R0L8C8	Lamina-associated polypeptide 2, isoforms beta/gamma (Fragment)	−0.302	0.607	−0.190	−0.142	0.969 **
R0LDS8	Mitochondrial proton/calcium exchanger protein (Fragment)	0.587	−0.672	0.539	0.450	−0.918 **
R0LG97	Dystrophin (Fragment)	−0.311	0.150	−0.642	−0.906 *	0.486
R0LPZ6	protein disulfide-isomerase (Fragment)	0.350	−0.257	0.498	0.822 *	−0.628
R0M8V6	Small ribosomal subunit protein mS38 (Fragment)	−0.557	0.639	−0.311	−0.307	0.956 **
U3I6H8	Laminin subunit gamma 1	−0.395	0.588	−0.366	−0.397	0.939 **
U3J8M4	Microtubule-associated protein	0.533	−0.689	0.337	0.280	−0.951 **

** *p* < 0.01; * *p* < 0.05.

**Table 4:** The expression and relative quantitative value of significantly correlated DEPs.

Protein	Relative Quantitative Value	
	Thigh Muscle (T)	Breast Muscle (X)
A0A493T986	23,629.41 ± 3678.78 ^b^	37,766.80 ± 2474.14 ^a^
A0A8B9SIU4	117,646.88 ± 2808.37 ^a^	95,333.83 ± 12,282.66 ^b^
A0A8B9STW6	6389.15 ± 513.74 ^b^	8179.67 ± 894.35 ^a^
A0A8B9TGK7	75,460.97 ± 12,741.76 ^a^	53,305.82 ± 4786.62 ^b^
A0A8B9TXS4	60,017.76 ± 6211.44 ^a^	38,633.88 ± 6553.00 ^b^
P19140	2,161,003.06 ± 96,743.83 ^b^	2,647,234.80 ± 250,263.90 ^a^
R0KF46	160,957.51 ± 3922.00 ^b^	226,767.17 ± 13,007.96 ^a^
R0LNL3	27,352.48 ± 1918.87 ^a^	18,187.82 ± 2529.56 ^b^
A0A493SUY8	2,658,794.72 ± 898,305.52 ^b^	5,450,842.91 ± 588,330.49 ^a^
A0A493SY37	26,516.16 ± 3106.40 ^b^	46,789.27 ± 6880.12 ^a^
A0A493SYB4	31,671.87 ± 16,626.34 ^b^	70,919.41 ± 13,447.53 ^a^
A0A493SYW9	276,359.20 ± 147,195.04 ^b^	840,661.65 ± 143,235.32 ^a^
A0A493T2Z7	38,858.08 ± 10,829.14 ^b^	74,619.64 ± 12,430.43 ^a^
A0A493TGA7	5563.71 ± 2362.75 ^b^	13,629.51 ± 3979.93 ^a^
A0A493TM94	45,039.22 ± 3005.91 ^b^	62,143.52 ± 6798.39 ^a^
A0A493U353	197,729.17 ± 38,556.37 ^b^	450,469.66 ± 112,562.17 ^a^
A0A8B9QQD8	62,867.57 ± 14,187.69 ^b^	108,785.90 ± 9467.38 ^a^
A0A8B9SJ62	997,289.96 ± 352,412.73 ^b^	2,096,673.55 ± 168,803.28 ^a^
A0A8B9TJ91	104,092.87 ± 43,913.76 ^b^	819,254.78 ± 114,328.27 ^a^
A0A8B9TP15	62,862.36 ± 6290.75 ^a^	35,776.21 ± 7543.99 ^b^
R0KUY7	48,546.55 ± 5548.37 ^a^	27,687.88 ± 5476.48 ^b^
R0L8C8	33,290.00 ± 4054.50 ^b^	55,844.39 ± 11,665.92 ^a^
R0LDS8	60,152.90 ± 7192.95 ^a^	30,559.12 ± 3609.13 ^b^
R0LG97	37,211.53 ± 2547.02 ^b^	48,377.48 ± 5152.38 ^a^
R0LPZ6	51,503.11 ± 745.30 ^a^	32,500.38 ± 5948.77 ^b^
R0M8V6	14,328.20 ± 6638.90 ^b^	33,238.78 ± 4767.94 ^a^
U3I6H8	112,689.43 ± 9336.91 ^b^	188,517.32 ± 16,328.81 ^a^
U3J8M4	56,334.78 ± 7872.86 ^a^	33,772.94 ± 5604.63 ^b^

a,b: different superscripts in the same row indicate a significant difference (*p* < 0.05).

**Table 5:** Pearson’s correlation analysis between differentially expressed proteins (DEPs) and meat quality traits of Sansui ducks at different ages.

Protein	Description	a*	b*	L*	pH	Shear Force
A0A493TG46	F-actin-capping protein subunit beta	0.663	0.743 *	−0.918 **	−0.743 *	0.746 *
A0A493TIN0	LSM7 homolog, U6 small nuclear RNA and mRNA degradation associated	0.697	0.727 *	−0.824*	−0.764 *	0.756 *
A0A493TJU7	Vacuolar protein sorting 13 homolog A	0.717 *	0.739 *	−0.858 **	−0.750 *	0.766 *
A0A493TWK1	bis(5-adenosyl)-triphosphatase	−0.680	−0.746 *	0.915 **	0.730 *	−0.771 *
A0A8B9SHF6	Glutathione S-transferase	−0.724 *	−0.636	0.810 *	0.779 *	−0.676
A0A8B9SIY9	Histidine-rich glycoprotein	0.717 *	0.538	−0.769 *	−0.725 *	0.624
A0A8B9T8F1	rRNA 2-O-methyltransferase fibrillarin	0.609	0.841 **	−0.847 **	−0.710 *	0.840 **
A0A8B9TF28	Ubiquitin-associated domain-containing protein 1	0.736 *	0.641	−0.876 **	−0.731 *	0.700
A0A8B9TR61	O-acyl-ADP-ribose deacylase 1	0.724 *	0.646	−0.886 **	−0.726 *	0.703
A0A8B9ZCM2	E2 ubiquitin-conjugating enzyme	0.709 *	0.545	−0.763 *	−0.714 *	0.632
A0A8B9ZKB4	2-oxoglutarate dehydrogenase complex component E1	−0.634	−0.822 *	0.845 **	0.738 *	−0.807 *
R0JG91	Short-chain specific acyl-CoA dehydrogenase, mitochondrial (Fragment)	−0.628	−0.836 **	0.802 *	0.717 *	−0.830 *
R0JHH1	WD repeat-containing protein 82 (Fragment)	0.677	0.640	−0.868 **	−0.795 *	0.664
R0JVD4	Bullous pemphigoid antigen 1, isoforms 6/9/10 (Fragment)	−0.608	−0.749 *	0.837 **	0.793 *	−0.755 *
R0K6B5	Alkylglycerone-phosphate synthase (Fragment)	0.742 *	0.649	−0.844 **	−0.741 *	0.681
R0LIV7	Mitochondrial pyruvate carrier (Fragment)	−0.711 *	−0.701	0.875 **	0.755 *	−0.720 *
R0LML0	Galactose mutarotase (Fragment)	−0.704	−0.710 *	0.895 **	0.733 *	−0.722 *
R0LNK7	Nodal modulator 2 (Fragment)	0.661	0.798 *	−0.781 *	−0.716 *	0.812 *

** *p* < 0.01; * *p* < 0.05.

**Table 6:** The expression and relative quantitative value of significantly correlated DEPs in different age groups.

Protein	Relative Quantitative Value		Up or Down
	D90	D468	D90 vs. D468
A0A493TG46	206,774.73 ± 21,992.63 ^b^	257,191.17 ± 18,169.73 ^a^	up
A0A493TIN0	25,396.78 ± 3505.68 ^b^	33,399.35 ± 2495.93 ^a^	up
A0A493TJU7	20,394.24 ± 1961.91 ^b^	25,382.50 ± 1535.21 ^a^	up
A0A493TWK1	2760.78 ± 284.07 ^a^	1859.90 ± 325.05 ^b^	down
A0A8B9SHF6	131,597.65 ± 25,022.85 ^a^	63,382.37 ± 32,425.90 ^b^	down
A0A8B9SIY9	11,215.36 ± 5738.26 ^b^	27,314.19 ± 8124.77 ^a^	up
A0A8B9T8F1	18,593.60 ± 4860.21 ^b^	27,948.02 ± 1850.34 ^a^	up
A0A8B9TF28	20,950.20 ± 2082.60 ^b^	30,660.24 ± 4256.40 ^a^	up
A0A8B9TR61	140,592.86 ± 11,022.55 ^b^	194,423.47 ± 24,004.08 ^a^	up
A0A8B9ZCM2	37,552.87 ± 3286.30 ^b^	45,982.10 ± 4055.90 ^a^	up
A0A8B9ZKB4	263,408.81 ± 57,212.95 ^a^	164,432.33 ± 17,534.20 ^b^	down
R0JG91	224,909.29 ± 50,278.82 ^a^	140,121.17 ± 9186.58 ^b^	down
R0JHH1	22,018.85 ± 4033.71 ^b^	33,068.83 ± 5556.74 ^a^	up
R0JVD4	18,576.76 ± 1941.04 ^a^	14,754.28 ± 1281.13 ^b^	down
R0K6B5	9573.39 ± 2231.31 ^b^	15,595.60 ± 2820.95 ^a^	up
R0LIV7	33,205.28 ± 6386.85 ^a^	17,530.04 ± 6132.00 ^b^	down
R0LML0	13,732.04 ± 2390.49 ^a^	8292.09 ± 2041.06 ^b^	down
R0LNK7	15,070.45 ± 2923.60 ^b^	20,334.21 ± 838.31 ^a^	up

a,b: different superscripts in the same row indicate a significant difference (*p* < 0.05); up indicates upregulation of expression, whereas down indicates downregulation of expression.

**Table 7:** Differential metabolite screening results.

Compared Samples	Num. of Total Ident.	Num. of Total Sig.	Num. of Sig. Up	Num. of Sig. Down
D90X vs. D468X pos	1094	260	197	63
D90T vs. D468T pos	1094	208	89	119
D90T vs. D90X pos	1094	280	44	236
D468T vs. D468X pos	1094	197	99	98
D90X vs. D468X neg	721	159	105	54
D90T vs. D468T neg	721	136	22	114
D90T vs. D90X neg	721	204	23	181
D468T vs. D468X neg	721	141	53	88

**Table 8:** Pearson’s correlation between DEMs and quality traits of duck meat in different muscle parts.

Metabolites	a*	b*	L*	pH	Shear Force	Up or Down
						T vs. X
Histidylmethionine	0.11	0.10	−0.41	0.19	−0.60 *	down
(6Z,9Z)-Hexadecadienoylcarnitine	−0.12	0.25	−0.68 **	0.19	−0.53 *	down
Phenylalanylalanine	0.31	−0.14	−0.12	0.14	−0.56 *	down
Val Ile	0.26	−0.05	−0.17	0.07	−0.59 *	down
DL-tert-Leucine	0.36	−0.09	−0.12	0.11	−0.55 *	down
Leu-Ala-Gly	0.24	0.00	−0.24	0.10	−0.59 *	down
DL-Leucyl-DL-phenylalanine	0.21	−0.01	−0.21	0.07	−0.59 *	down
Hemorphin-4	0.05	0.08	−0.36	0.13	−0.66 **	down
L-Valyl-L-phenylalanine	0.27	−0.06	−0.20	0.03	−0.64 *	down
H-Ser-Leu-OH	0.40	−0.22	−0.04	−0.05	−0.61 *	down
L-Leucyl-L-Valine	0.28	−0.07	−0.16	0.07	−0.59 *	down
Ala-Phe	0.32	−0.15	−0.11	0.09	−0.56 *	down
H-Thr-Phe-OH	0.23	−0.03	−0.19	0.08	−0.58 *	down
H-ILE-LEU-OH	0.32	−0.12	−0.12	0.03	−0.60 *	down
Val Trp	0.14	−0.04	−0.30	0.14	−0.60 *	down
*N*-decanoylglycine	−0.02	−0.08	0.17	0.84 **	0.54 *	down
Glycyl-L-leucine	0.32	−0.08	−0.15	0.12	−0.56 *	down
1-(3-Aminopropyl)-4-aminobutanal	0.35	−0.11	−0.13	0.10	−0.58 *	down
Val Val Ile	−0.20	0.15	−0.39	0.17	−0.54 *	down

** *p* < 0.01; * *p* < 0.05. T: thigh muscle; X: pectoralis muscle.

**Table 9:** Pearson’s correlation between DEMs and quality traits of duck meat at different ages.

Metabolites	a*	b*	L*	pH	Shear Force	Up or Down
						90d vs. 468d
(2R,4R)-4-Aminopyrrolidine-2,4-dicarboxylic acid	0.16	0.40	−0.55 *	0.92 **	0.60 *	down
Pro Ile	0.03	0.40	−0.50	0.80 **	0.53 *	down
beta-Alanyl-L-arginine	0.26	−0.35	0.55 *	−0.73 **	−0.55 *	up
PC(18:1(11Z)/18:1(12Z)-2OH(9,10))	−0.51	−0.02	0.40	−0.69 **	−0.54 *	up
4-Keto-clonostachydiol	0.26	0.27	−0.33	0.90 **	0.76 **	down
2-hydroxytridecanoic acid	0.34	0.20	−0.54 *	0.90 **	0.65 *	down
Ethosuximide	0.16	0.30	−0.57 *	0.87 **	0.57 *	up
2,5-Dimethyl-1,4-dithiane-2,5-diol	0.14	0.29	−0.53	0.86 **	0.61 *	up
9,10-Dihydroxystearate	0.09	0.14	−0.51	0.78 **	0.53 *	up
Inosinic acid	0.25	0.33	−0.57 *	0.82 **	0.53 *	down

** *p* < 0.01; * *p* < 0.05.

**Table 10:** The list of the specific proteins and metabolites involved in the shared pathways between metabolomics and proteomics from different age groups.

Pathway		Names	90X vs. 468X			90T vs. 468T		
			log2FC	*p*-Value	Trend	log2FC	*p*-Value	Trend
Lysine degradation	Protein	A0A8B9TTI1	0.359	0.026	↑	−0.271	0.019	↓
		R0JRM6	0.287	0.020	↑	−0.279	0.025	↓
	Metabolites	L-Lysine	1.061	0.012	↑	1.151	0.010	↑
		L-Pipecolic acid	0.995	0.012	↑	1.053	0.011	↑
Butanoate metabolism	Protein	A0A8B9TTI1	0.359	0.026	↑	−0.271	0.019	↓
	Metabolites	gamma-Aminobutyric acid	1.002	0.016	↑	0.950	0.038	↑
2-Oxocarboxylic acid metabolism	Protein	A0A8B9SQI5	0.764	0.047	↑	0.610	0.046	↑
	Metabolites	Palmitoleic acid	0.620	0.034	↑	1.093	0.003	↑
		L-Ornithine	0.937	0.035	↑	1.096	0.046	↑

**Table 11:** The list of the specific proteins and metabolites involved in the shared pathways between metabolomics and proteomics from different muscle parts.

Pathway		Names	90X vs. 90T			468X vs. 468T		
			log2FC	*p*-Value	Trend	log2FC	*p*-Value	Trend
ABC transporters	Protein	R0JXJ3	−0.358	0.044	↓	−0.823	0.001	↓
	Metabolites	Choline	0.987	0.007	↑	0.813	0.013	↑
		L-Glutamine	−1.366	0.018	↓	0.898	0.014	↑

## Data Availability

The data presented in this study are available on request from the corresponding author.

## Supplementary Materials

The following supporting information can be downloaded at: https://www.mdpi.com/article/10.3390/ani15192773/s1, Table S1: Ingredient and nutrient levels of the commercial diets in Sansui ducks (<14 days). Table S2: Ingredient and nutrient levels of the commercial diets in Sansui ducks (>15 days); Figure S1: Gene Ontology classification. Figure S2: Protein-protein interaction network analysis.
