# Supplementary material for: Integrated Proteomics and Metabolomics Profiling Unravels Molecular Mechanisms Underlying Postmortem Meat Quality Between Two Ages and Muscle Types in Sansui Duck
Source: Animals (Basel). 2025 Sep 23;15(19):2773. doi: 10.3390/ani15192773 (PMC12523418; doi:10.3390/ani15192773)
Supplement: Supplementary file 1 [file animals-15-02773-s001.zip › animals-3851882-supplementary.pdf]

The compound feed was sourced from Guilin Liyuan Grain, Oil and Feed Co., Ltd. Due to commercial confidentiality, the specific formulation ratios are not disclosed by the company.

Table S1. Ingredient and nutrient levels of the commercial diets in Sansui ducks(<14days).

Items		
Ingredient	Nutrion levels(%)	
Corn	Crude protein	17.0-20.0
Soybean meal	Crude fibre	≤6.0
Wheat	Calcium chloride	0.30-0.80
Sorghum	Crude ash	≤12.0
fish meal	Methionine	0.22-0.90
$CaHPO_4$	Ca	0.80-1.50
Limestone	P	≥0.50
Nacl		
Premix[1]		

[1]Contains L-lysine sulphate, DL-methionine, vitamin A, vitamin D_3, vitamin E, vitamin K_3, copper sulphate, ferrous sulphate, zinc sulphate, calcium propionate, etc.

Table S2. Ingredient and nutrient levels of the commercial diets in Sansui ducks(>15days).

Items		
Ingredient	Nutrion levels(%)	
Corn	Crude protein	≥15.0
Soybean meal	Crude fibre	≤8.0
Wheat	Calcium chloride	0.30-0.80
Sorghum	Crude ash	≤10.0
fish meal	Methionine	0.15-0.90
$CaHPO_4$	Ca	0.80-1.50
Limestone	P	≥0.50

Nacl	
Premix[1]	

[1]Contains L-lysine sulphate, DL-methionine, vitamin A, vitamin D_3, vitamin E, vitamin K_3, copper sulphate, ferrous sulphate, zinc sulphate, calcium propionate, etc.

A

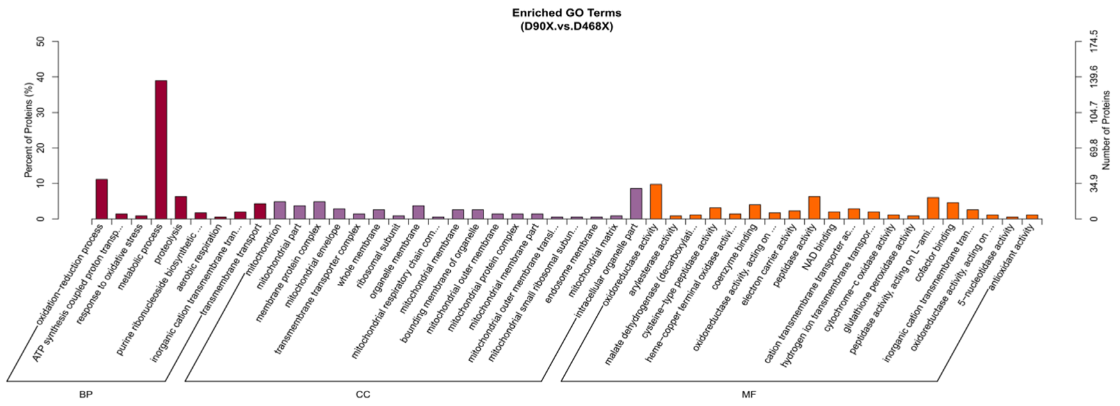

B

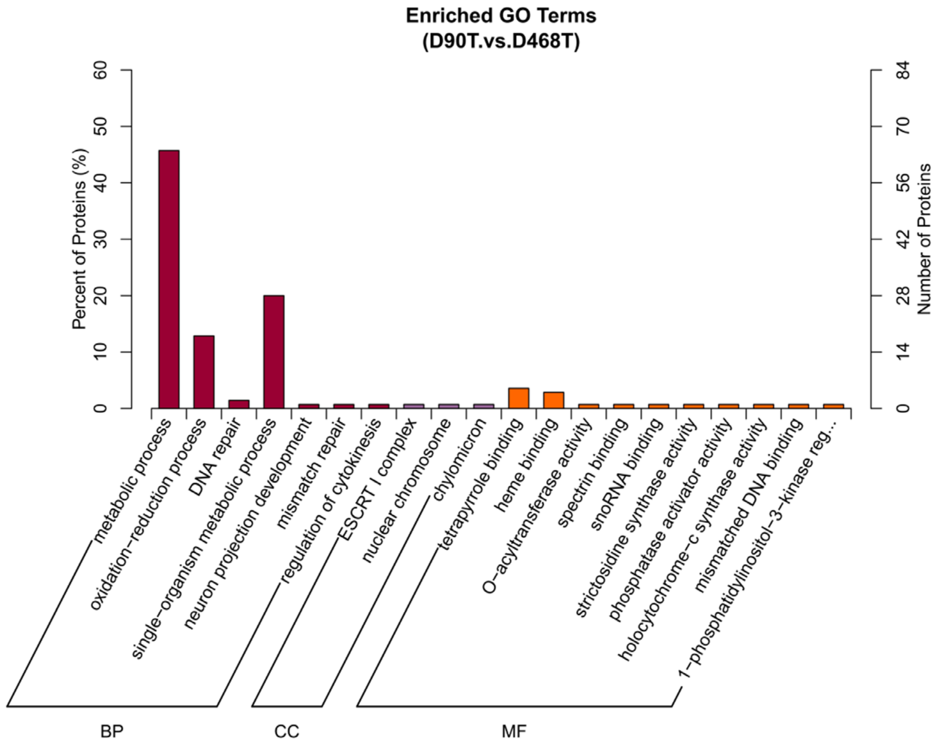

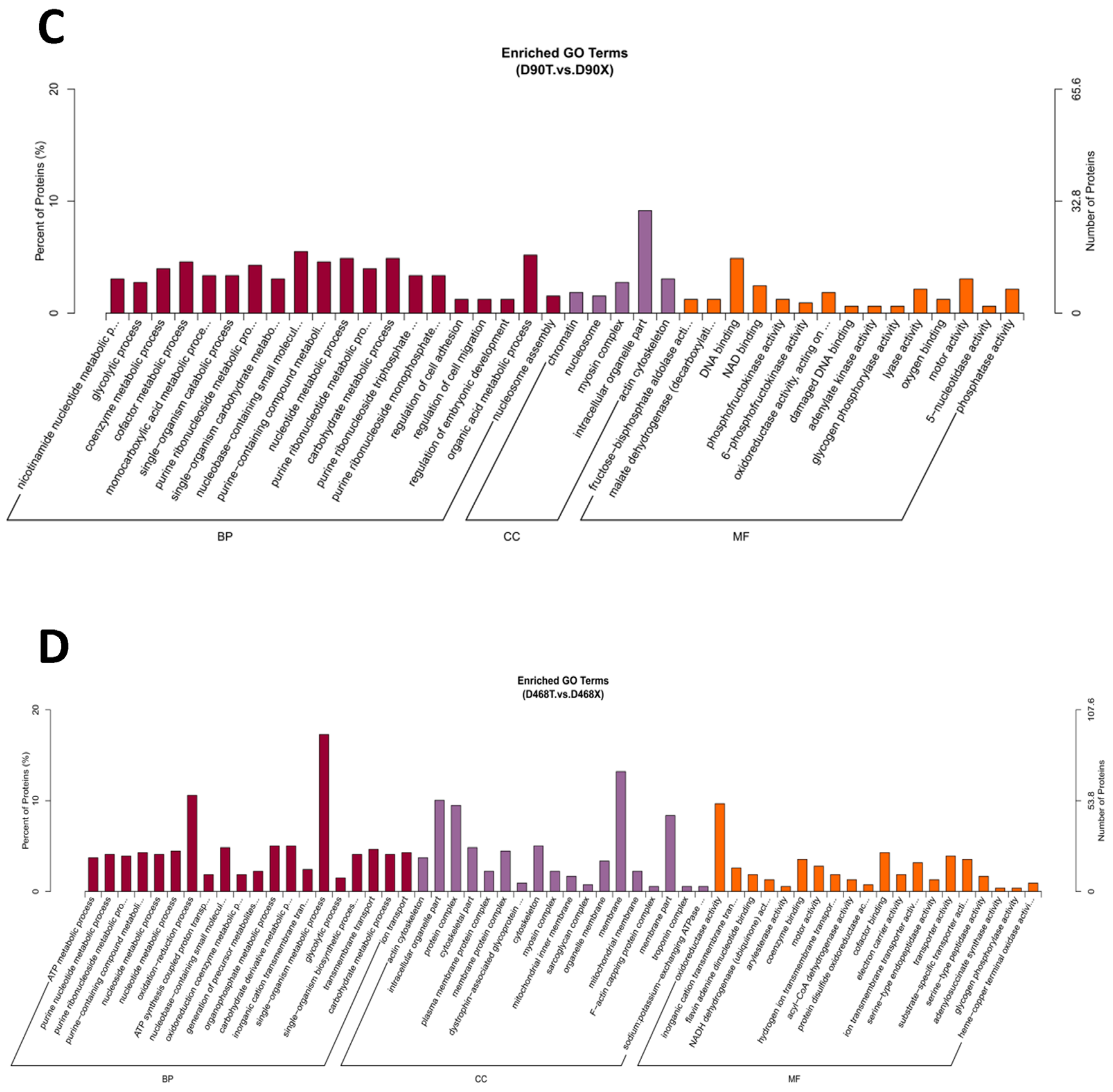

Figure S1. Gene Ontology classification. (A): D90X vs D468X; (B):D90T vs D468T; (C): D90X vs D90T; (D): D468X vs D468T.

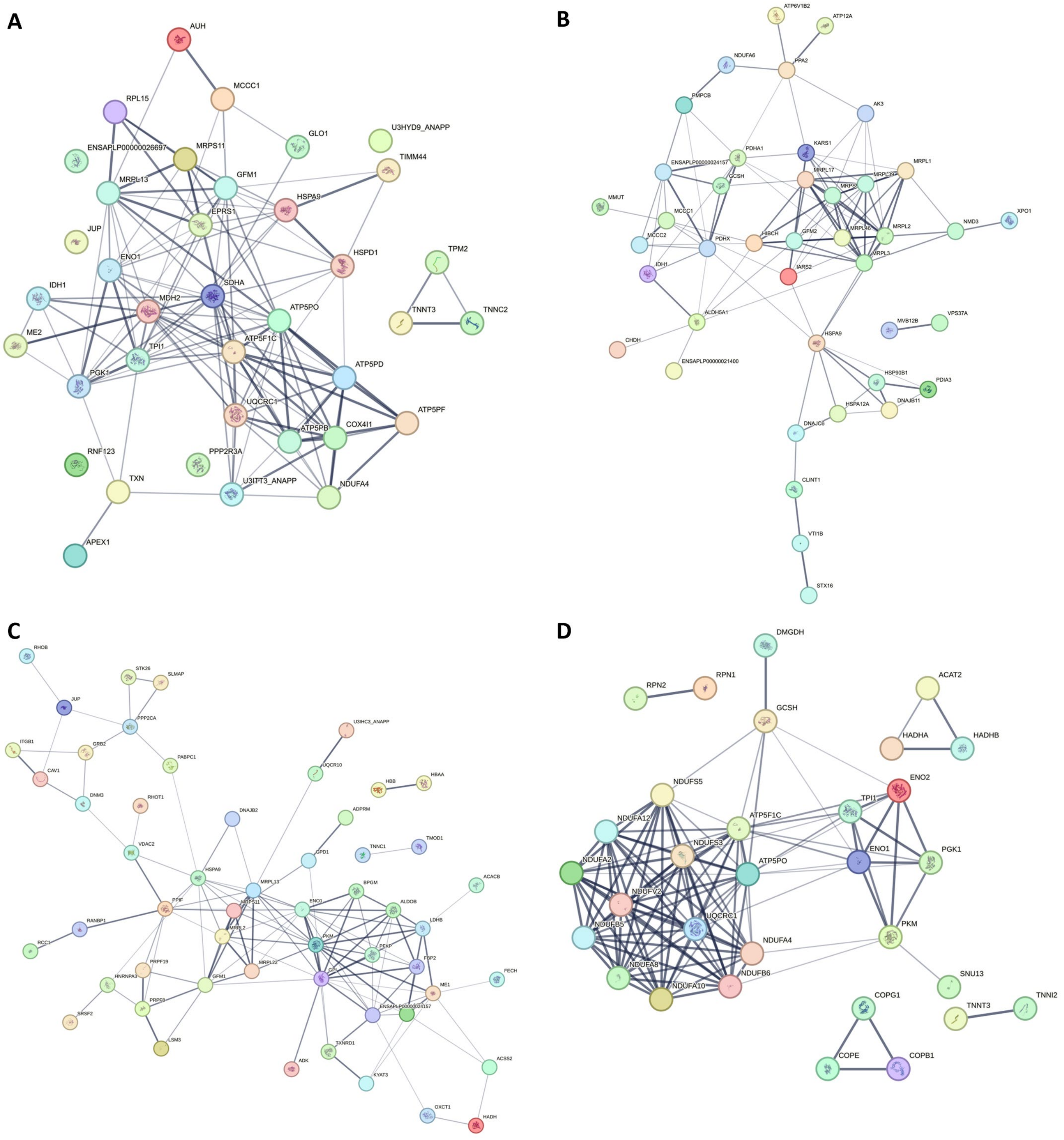

Figure S2. Protein-protein interaction network analysis. (A): D90X vs D468X; (B):D90T vs D468T; (C): D90X vs D90T; (D): D468X vs D468T.
